# Initial Experience Combining Negative Pressure Wound Therapy With Autologous Skin Cell Suspension and Meshed Autografts

**DOI:** 10.1093/jbcr/irab075

**Published:** 2021-04-27

**Authors:** Bonnie C Carney, Laura S Johnson, Jeffrey W Shupp, Taryn E Travis

**Affiliations:** 1 Department of Biochemistry and Molecular & Cellular Biology, Georgetown University Medical Center, Washington, District of Columbia, USA; 2 Firefighters’ Burn and Surgical Research Laboratory, MedStar Health Research Institute, Washington, District of Columbia, USA; 3 Department of Surgery, Georgetown University School of Medicine, Washington, District of Columbia, USA; 4 The Burn Center, Department of Surgery, MedStar Washington Hospital Center, Washington, District of Columbia, USA

## Abstract

The success of autologous split-thickness skin grafts (STSGs) in the treatment of full-thickness burns is often dependent on the dressing used to secure it. Tie-over bolsters have been used traditionally; however, they can be uncomfortable for patients and preclude grafting large areas in one definitive operation. Negative pressure wound therapy (NPWT) is used as an alternative to bolster dressings and may afford additional wound healing benefits. In our center, NPWT has become the dressing of choice for securing STSGs. While the RECELL^®^ system is being used in conjunction with STSGs, it is currently unknown whether autologous skin cell suspensions (ASCS) can be used with NPWT. This report is a retrospective chart review of nine patients treated in this manner. All wounds were almost completely re-epithelialized within 14 days, and their healing was as expected. Wound healing trajectories are shown. There were no significant complications in these patients. This dressing technique can be considered as an option when using ASCS and widely meshed STSG.

Burn injury is a significant healthcare burden in the United States and internationally. Burns can result in both functional and psychosocial complications for patients.^1^ The standard of care for full-thickness burns that cause irreparable damage to the entire cutis is early excision of the devitalized tissue and ultimately providing coverage to the excised wound bed with an autologous graft.^2^ Skin grafts may take the form of temporary agents such as biological dressings or xenografts^3^ or can be permanent as is the case for autologous skin grafts. Even when non-autologous tissues are used as temporizing agents, ultimately autograft is necessary for permanent coverage in cases of full-thickness injury. When total body surface area (TBSA) burn injuries are large, the area of donor site needed to produce necessary split-thickness skin grafts (STSGs) can be high, resulting in additional excisional wounds that require time and care for healing. These large TBSA injuries have the added challenge of limited healthy skin from which to harvest these STSGs. Hence, full wound closure is often delayed until donor site wounds can heal and be re-harvested. An additional consideration is that of donor site morbidity, which frequently can result in hypertrophic scarring with associated pain and itch.^4^

In the setting of skin grafting, the quality of a secondary dressing is extremely important, given the high potential for skin graft failure due to shearing, hematoma, and seroma accumulation. When extremities are grafted, a circumferential compression dressing is often a simple, effective way to hold a graft in place. When grafting areas that cannot be circumferentially wrapped, such as a torso, axilla, or neck, tie-over bolsters are often employed for graft fixation.^5^ Tie-over bolsters are created with a perimeter of robust sutures around the grafted area which are then tied to one another in multiple directions over a fluffed dry dressing to apply evenly dispersed pressure over the area of STSG. They can be tedious to create and painful for the patient. In addition, in the case of a very large wound, such as one encompassing the torso, axilla, and arm, tie-over bolsters are not possible to create, necessitating more than one operation. With the advent of negative pressure wound therapy (NPWT), the technique has commonly been adapted to affix STSG with or without primary dressings. In most cases where a perimeter of intact skin exists, many favor using NPWT as an alternative to bolsters or compression wraps. This approach is often less painful for patients, reduces fluid around the graft, and allows for the grafting of very large areas in one operation. NPWT has an added benefit of providing some degree of joint immobilization (enough to prevent shear) negating the need for postoperative splints and casts often allowing for improved early mobilization and participation with rehabilitation therapy.^6–8^ Additionally, various studies have cited improved engraftment of STSG with NPWT when compared with traditional bolsters.^5,9,10^

Widely meshed autograft has been employed for decades to maximize the amount of coverage one can achieve with a small available donor site in autografting of large TBSA full-thickness injuries. The downsides to this technique include unfavorable cosmetic outcomes, prolonged time to healing, as well as worsened hypertrophic scarring due to the large interstices left in a widely meshed skin graft. The RECELL^®^ system, used in combination with widely meshed autografts, has been shown to reduce donor site size with comparable healing compared to small meshing ratios.^11^ It is a commercially available technique to minimize donor site requirements and contribute to faster healing at widely meshed autografted sites. The device allows for expansion ratios of up to 80:1 compared to traditional meshed skin graft expansion systems which are typically limited to a maximum mesh ratio of 6:1. Commonly used expansion ratios are 2:1 and 3:1, with rare expansion to the large 6:1 ratio unless in the case of absolute necessity in massive burns (>80% TBSA). The RECELL^®^ system generates an autologous skin cell suspension (ASCS) that contains keratinocytes, melanocytes, and fibroblasts.^12^ The suspensions can be sprayed over a widely meshed skin graft (eg, 4:1) to allow it to heal in a similar manner to a smaller meshed skin graft (eg, 3:1) and contributes to faster wound healing, resulting in a 32% smaller overall donor site requirement, while remaining cost-effective.^11,13,14^ The clinical trials for this device did not include NPWT as a dressing strategy for STSG. Given the growing preference in some centers for using NPWT and wish to continue to use ASCS in cases requiring large meshed STSG, an animal model was conducted to determine whether the two technologies were compatible.

Prior to any patients being treated using this technique, a preclinical porcine model of burn, excision, 4:1 meshed STSG, ASCS application, and NPWT was recently completed in our laboratory.^15^ NPWT was used as a bolster dressing and compared to the traditional tie-over bolster technique for securing STSGs plus ASCS. There was no difference in wound healing between the NPWT and non-NPWT-dressed wounds, and all wounds healed within 14 days with no hypertrophic scar. Hence, NPWT was determined to be an efficacious choice for securing widely meshed STSG sprayed with ASCS. After the completion of the animal study, this dressing technique was used with success in our patient population. The below case series is a report on our initial experience.

## CASE SERIES

### Overview

These data were collected as a retrospective chart review under an approved institutional review board protocol #00002961. The median age was 53 years (interquartile range [IQR] 37–69), median TBSA was 12.5% (IQR 8–18), and the patients were predominantly female (66.6%). Burn injuries were a result of scalds or flames and affected a variety of anatomic locations (Table 1).

**Table 1.** Age, sex, etiology of burn, and % TBSA

Patient No.	Age	Sex	Etiology of Burn	% TBSA	Burn Location
1	37	F	Flame plus accelerant	18	RUE, RLE, R torso
2	73	F	Scald, water	13	RUE, LUE, R torso
3	71	F	Scald, water	7	Posterior torso, buttocks
4	53	M	Scald, cooling protocol	20	Bilateral thighs
5	69	M	Scald, water	9	Torso, R thigh
6	26	F	Flame	18.5	Bilateral UE, bilateral LE
7	48	F	Flame	8	L palm, L LLE
8	64	F	Scald, water	7	LUE, L torso, LLE, lower abdomen, L hip
9	37	M	Scald, grease	12	Bilateral UE, bilateral hands, L neck, abdomen, and bilateral ankles and feet

*RUE*, right upper extremity; *RLE*, right lower extremity; *R*, right; *L*, left; *LUE*, left upper extremity; *LLE*, left lower extremity; *M*, male; *F*, female.

**Figure 1.**
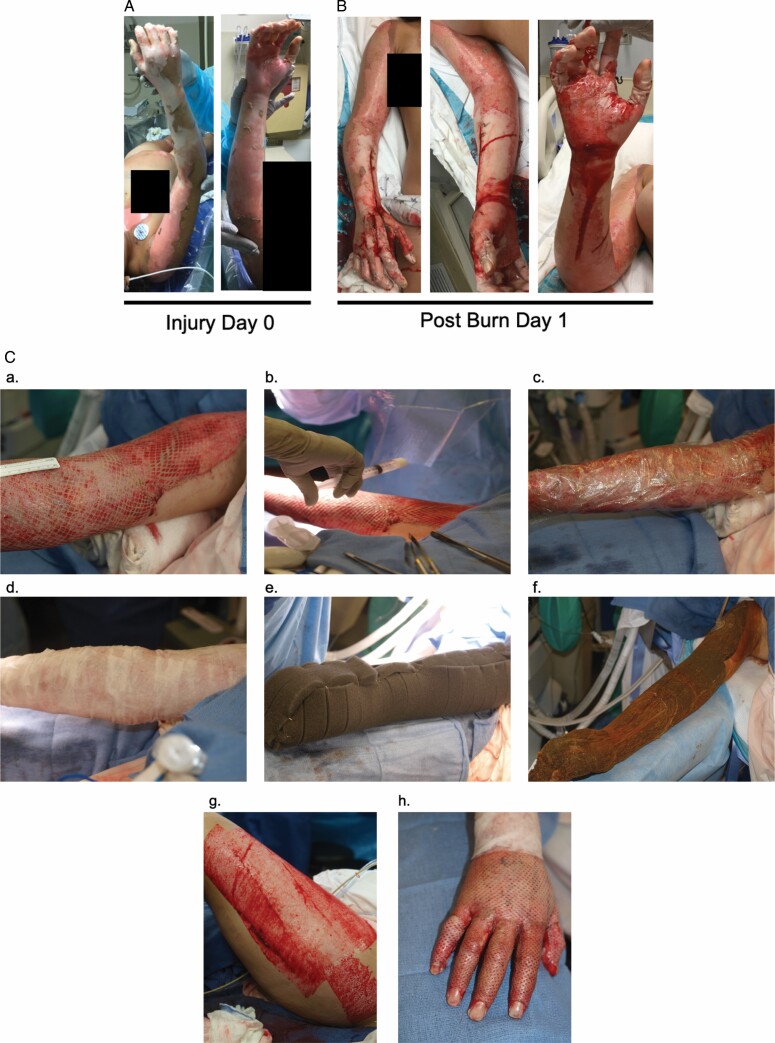

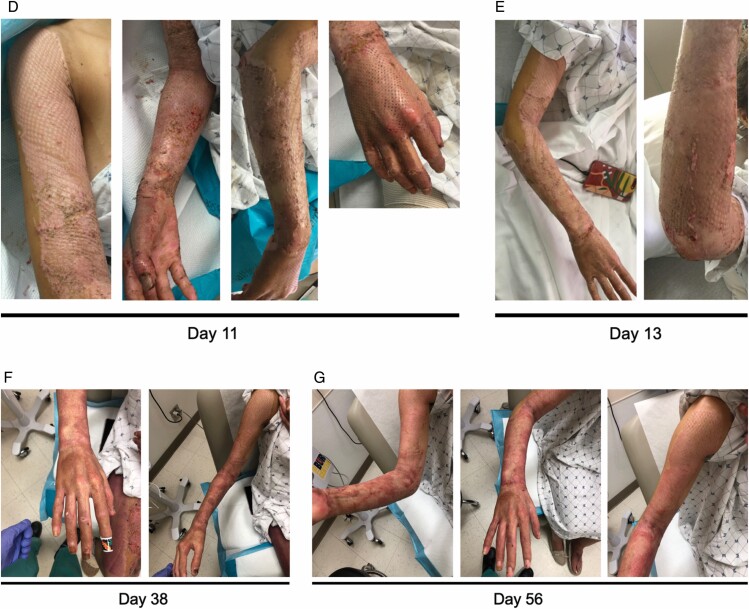
Patient 6 injury, treatment, and wound healing time course. Injury day 0 (A), postburn day 1 (B), and (C) intraoperative autografting: 3:1 meshed split-thickness skin graft is applied to a prepped wound bed (a). ASCS suspensions are sprayed over the meshed graft (b). Telfa clear is used as the primary dressing to promote adherence of the ASCS (c). Aquaphor-impregnated N-terface and fine-meshed gauze are used as a secondary dressing (d). NPWT sponges are applied (e). A seal is achieved with −125 mm Hg of pressure (f). Large donor site areas are required to cover large TBSA injuries (g). Small meshed ratios such as 1:1 are often utilized over functional areas such as the hand (h). Postoperative day 11 (D), day 13 (E), day 38 (F), and day 56 (G).

The usage of ASCS in this series of patients was chosen to reduce donor site area in patients with large injuries or in patients with smaller injuries but advanced age or poor overall health (Figure 1A and B). NPWT was chosen as the secondary dressing in these patients.

**Figure 2.**
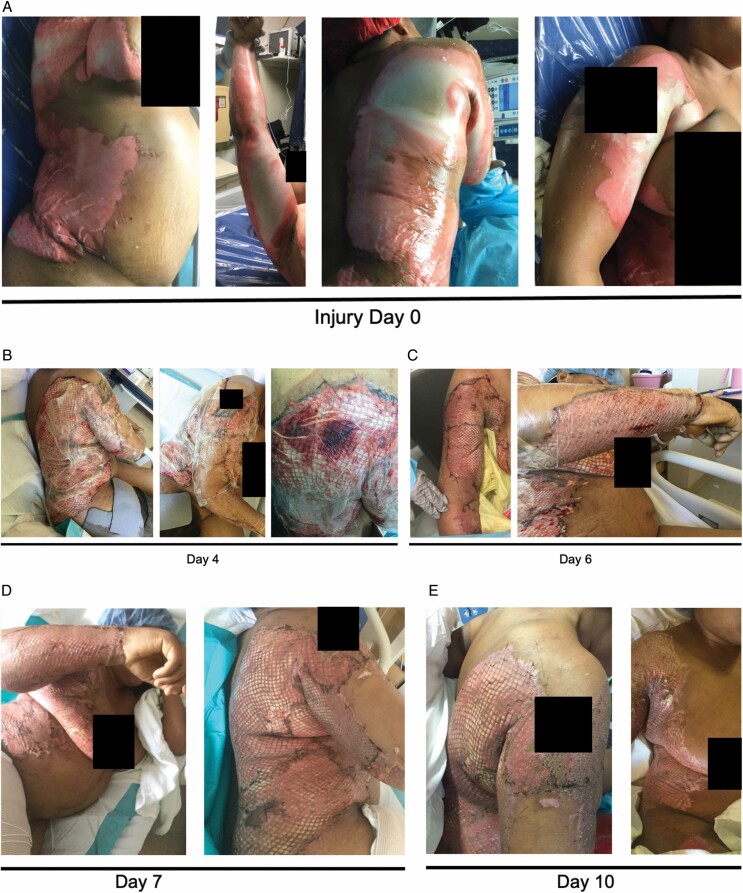

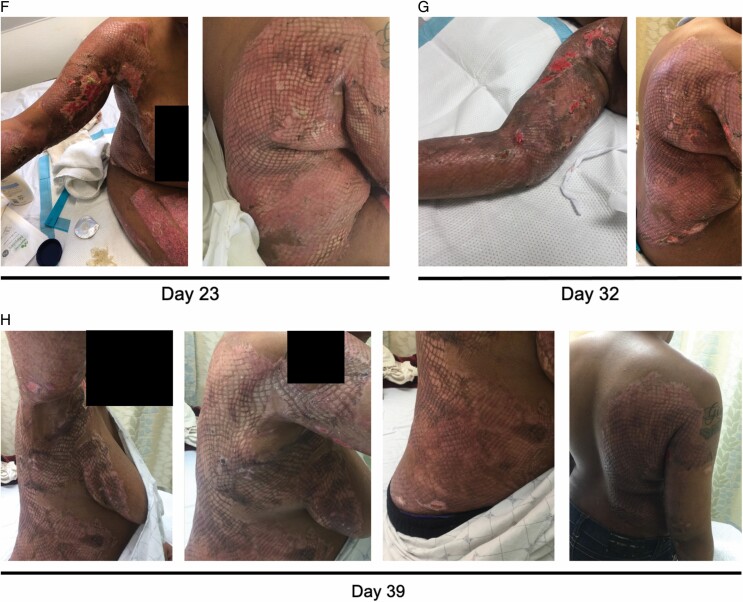
Patient 1 injury, treatment, and wound healing time course. Injury day 0 (A), postoperative day 4 (B), day 6 (C), day 7 (D), day 10 (E), day 23 (F), day 32 (G), and day 39 (H).

**Figure 3.**
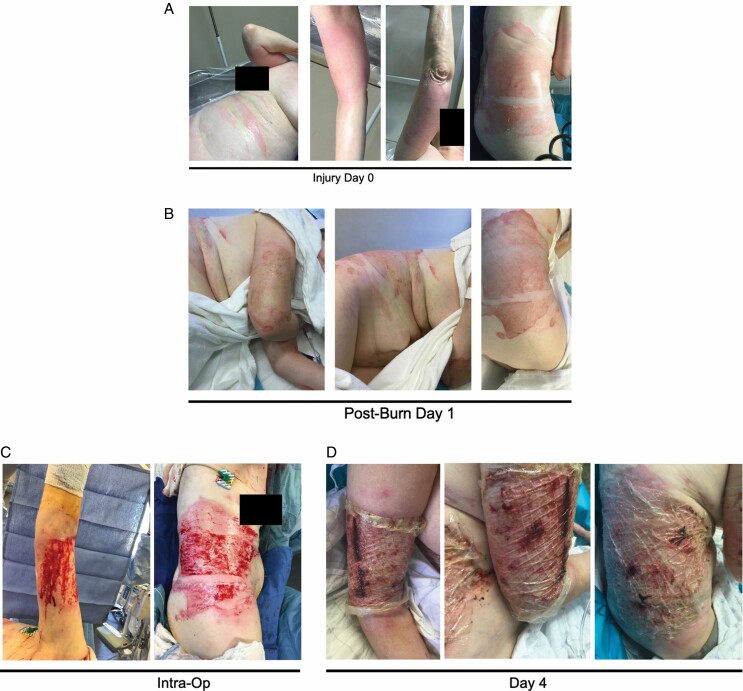

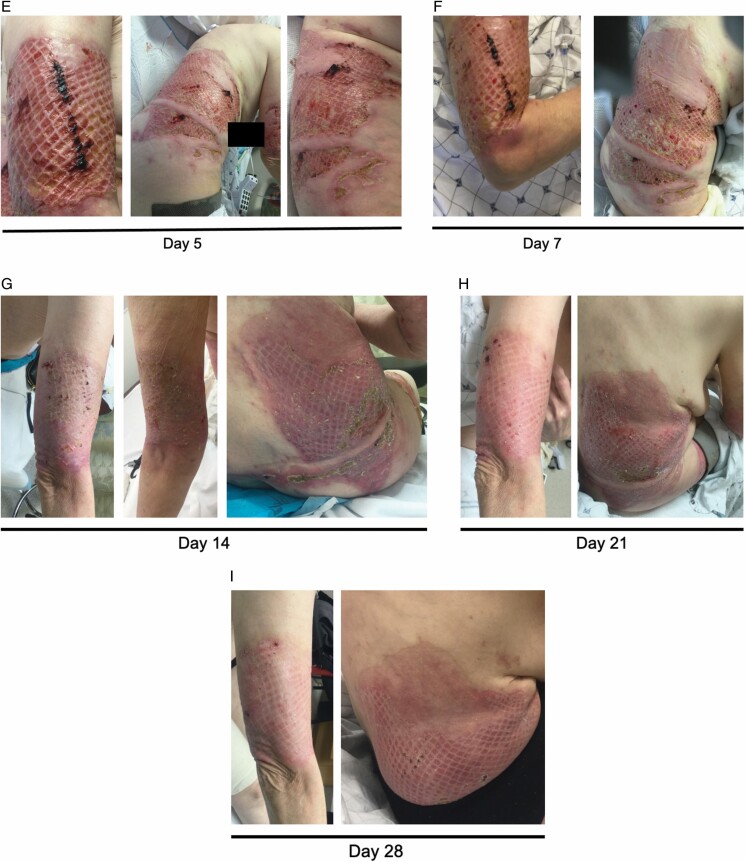
Patient 2 injury, treatment, and wound healing time course. Injury day 0 (A), postburn day 1 (B), intraoperative (C), day 4 (D), day 5 (E), day 7 (F), day 14 (G), day 21 (H), and day 28 (I).

Following tangential excision, all patients had STSG obtained with a cordless dermatome (Humeca, Woodstock, GA) at a depth of 0.008 to 0.012′′. Depending on the %TBSA to be covered, age of the patient, donor site availability, and medical comorbidities, the STSG was meshed either 4:1 (patients 1, 2, 3, and 5) or 3:1 (patients 4, 6, 7, 8, and 9), with the larger mesh size being chosen to further reduce donor site morbidity when needed. The graft was laid onto the prepared wound bed and tacked into place with 5-0 chromic sutures. ASCS was sprayed over the mSTSG and Telfa Clear (Covidien, Dublin, Ireland) was immediately overlayed as the primary dressing. Aquaphor-impregnated Conformant-2 (Smith + Nephew, London, UK) non-adherent dressing was then applied followed by Aquaphor-impregnated fine mesh gauze (DeRoyal, Powell, TN). The last component of the dressing was an NPWT device (KCI, San Antonio, TX) set to −125 mm Hg, continuous pressure with medium intensity. Adhesive seal was achieved using Ioban^TM^ adhesive drape (3M, St. Paul, MN) and benzoin topical adhesive where necessary (skin creases and difficult to conform areas; Figure 1C). All dressings remained in place for 3 days. On postoperative day 3, the NPWT was removed along with the Conformant-2 and fine mesh gauze. The primary dressing (Telfa Clear) remained in place until postoperative day 5. On postoperative day 5, Telfa Clear was removed, patients began daily showers with soap and water, and grafted sites were covered with xeroform gauze until epithelialization was complete. Per usual management, patients returned for an initial outpatient visit within 7 to 10 days from discharge. Patients were prescribed custom compression garments and referred for outpatient burn rehabilitation therapy. Three representative examples of patient initial injury, operative management, and wound healing trajectory are shown (Figures 1–3).

**Figure 4.**
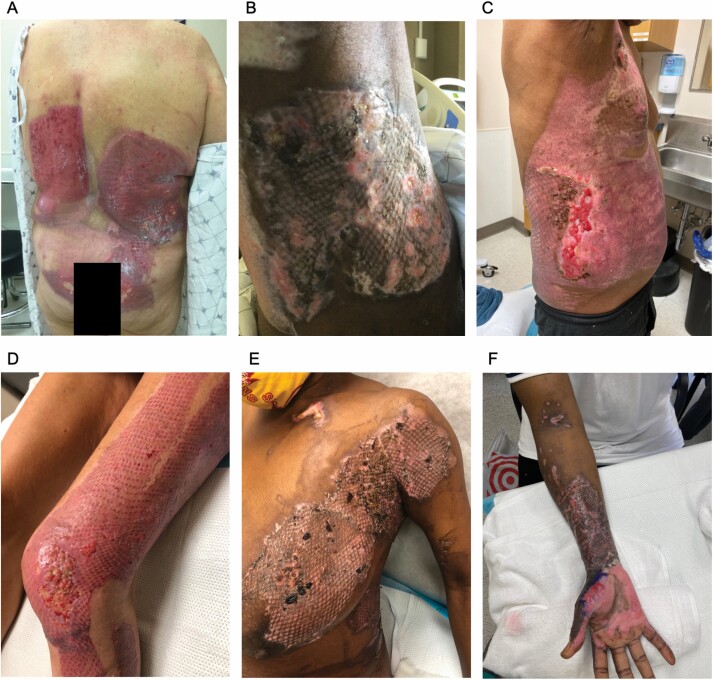
Final re-epithelialization results for all patients not previously shown. Patient 3 at day 13 post-grafting (A), patient 4 at day 14 post-grafting (B), patient 5 day 17 post-grafting (C), patient 7 day 20 post-grafting (D), patient 8 day 15 post-grafting (E), and patient 9 day 14 post-grafting (F).

There were no NPWT device failures requiring intervention to reachieve an adequate seal. Each patient followed a trajectory of expected wound healing at the sites that were treated with the above technique. Approximately 85% re-epithelialization was achieved in 8 of 9 patients by postoperative day 13. Patient 4 had approximately 75% re-epithelialization with some minor graft loss at day 14 (Figure 4). On their next follow-up visit, their wounds were closed.

There were no mortalities in this cohort of patients. Morbidities included minor graft loss (10–25% of the meshed autografted site that was treated with ASCS corresponding to Grade I graft loss on our institutional scale), resolved with local wound care and not requiring re-operation at that site.^16^ Additional morbidities include those related to the development of hypertrophic scar (functional limitations, pain, pruritis, reduced elasticity, stiffness, and thickness of scar), which are predictable and frequently develop regardless of dressing strategy.

## DISCUSSION

The RECELL^®^ system has been shown to be a useful adjunct to traditional widely meshed STSG in achieving wound closure while minimizing donor site size. Using NPWT to secure STSG coupled with ASCS had not been previously reported in the literature. It may seem paradoxical to spray cells and buffer containing potentially beneficial noncellular components, such as growth factors or anti-inflammatory cytokines, and then immediately apply an NPWT device. It is possible that the cells and buffer would be taken into the vacuum component of the sponge. Our preclinical porcine modeling testing this hypothesis showed noninferiority of NPWT, suggesting that the ASCS that were sprayed stayed in place, were not taken into the vacuum, and positively contributed to wound healing.^15^ The inherent limitation to this series was that we did not use a control tie-over bolster for direct comparison within each patient. We report based on our extensive experience as a site for all of the prior RECELL^®^ trials that the wound healing trajectories observed in this case series are similar to those achieved with tie-over bolsters in previous patients. Despite the similar healing, the advantages to using NPWT were harnessed, such as our ability to provide definitive closure over large TBSA injuries in one operative intervention in multiple patients, and the opportunity for earlier mobilization in these patients without graft shearing. Lastly, while not related to NPWT, due to the fact that these surgical procedures were conducted in patients with relatively large TBSA, there was a significant reduction in overall donor site area requirement compared to had we not used the RECELL^®^ technique. To the best of our knowledge, this is the first report of combining the technologies of RECELL^®^ and NPWT in the treatment of full-thickness burn wounds. Ultimately, successful wound closure was achieved in this cohort of patients, and no disadvantages were noted in using NPWT to secure STSGs sprayed with ASCS.

## CONCLUSION

NPWT can be used in conjunction with RECELL^®^ when preferred as a means of securing meshed STSG.
